# Functional Outcome of Intraarticular Fracture of Distal Radius Managed by Volar Locking Plate

**DOI:** 10.7759/cureus.11271

**Published:** 2020-10-31

**Authors:** Masroor Ahmed, Naveed Ahmed, Sunil Kumar, Mukesh Kumar, Muhammad Bux, Ghulam Hussain

**Affiliations:** 1 Orthopedic Surgery, Shaheed Mohtarma Benazir Bhutto Medical College Lyari, Karachi, PAK; 2 Orthopedic Surgery, Khairpur Medical College, Khairpur, PAK; 3 Trauma and Orthopedic Surgery, Dow University of Health Sciences, Karachi, PAK; 4 Orthopedic Surgery, Begum Haji Yousuf Jamiyat Hospital, Karachi, PAK; 5 Orthopedic Surgery, Sheikh Zayed Taluka Headquarter Hospital, Thatta, PAK

**Keywords:** distal radius fracture, functional outcome, intraarticular fracture, volar locking plate

## Abstract

Objective

This study's main purpose is to determine the functional outcome of volar locking plates in the management of intraarticular fracture of the distal radius.

Methodology

This cross-sectional study was conducted from August 2016 to August 2019. Forty-nine patients with the intraarticular distal radius fractures managed by the volar locking plate were included in the study. Patients with open fractures, ipsilateral other limb injuries, polytrauma patients, pathological fractures, and patients having neurovascular injuries were excluded from the study. AO Classification was used to classify fractures. The fracture was approached through the volar approach and fixed by the volar locking plate. A modified mayo wrist score evaluated the functional outcome. All the data were recorded on predesigned performa, and Statistical Package for the Social Sciences (SPSS), version 20 (IBM Corp., Armonk, NY) was used to analyze the data.

Results

A total of 49 patients with intraarticular fractures of the distal radius were included in the study. The mean age of the patient was 37.20 ± 10.05 years. Out of 49 patients, 29 (59.2%) were males, and 20 (40.8%) were females. Union was achieved in almost all fractures except one case, which went into non-union despite the adequate initial reduction, and the mean time of union was 11.98 ± 1.64 weeks. With respect to the stratification of functional outcome very good and good functional outcome was achieved in 46 patients (93.8%) in both the groups, the satisfactory outcome was achieved in two (4.1%) cases and one patient had a bad outcome in which union was not achieved and went into non-union.

Conclusion

Open reduction and internal fixation of intraarticular fracture of the distal radius using a volar locking plate is a good option for managing these fractures as it provides stable fixation, and good to excellent outcomes can be achieved by using these plates.

## Introduction

Fractures of distal radius constitute about 16% of all and 74.5% of the forearm's fractures being managed at the emergency department [1]. Intraarticular involvement is one of the complex patterns and constitutes about 25% of such injuries [2]. It has got the bimodal distribution with increased incidence in old age due to osteoporosis and in the young population due to high-velocity injuries and outdoor activities [3]. Being the intraarticular, fracture of distal radius accurate reduction and stabilization is the challenge for these complicated injuries despite the controversies in treatment and rehabilitation of these injuries [4]. Volar/dorsal tilt, radial inclination, ulnar variance, and intraarticular step-off are the important factors to assess the severity of the injury and decide on the optimum treatment option for the fracture [5].

The benefits of using volar locking plates include direct fracture fragment reduction and stable fixation and early postoperative physiotherapy leading to the early return of range of motion and return to work [6-8]. The number of complications associated with volar locking plates is relatively low as compared to dorsal plating [9]. When comparing the volar locking plate with non-locking constructs biomechanically, it appears to be more stable and even holds the dorsally displaced fragment [10,11].

This study aims to find the functional outcome of volar locking plates in the management of intraarticular fracture of the distal radius.

## Materials and methods

This cross-sectional study consists of 49 adult patients with an average age of 37.20 ± 10.05 years with displaced intraarticular distal radius fracture who presented to our tertiary center from August 2016 to August 2019. Non-probability consecutive sampling techniques were used for sampling. All the patients with an intra-articular fracture of distal radius who consented to be part of the study, medically fit, and over the age of 20 years were included in the study. Patients with open fractures, ipsilateral other limb injuries, polytrauma patients, pathological fractures, and patients having neurovascular injuries were excluded from the study. The study was conducted after approval from the ethical review board.

AO Classification was used to classify fractures. The fracture was approached through the volar approach and fixed by the volar locking plate. The active-assisted movement was gradually started according to the patient's pain tolerance, followed by passive movement by the physiotherapist at four weeks. Patients were followed up at two weeks, six weeks, three months, and six months regularly. Union was assessed clinically and radiologically after six weeks of surgery. A modified mayo wrist score was used at the six months to evaluate the functional outcome. All the data were recorded on predesigned performa, and Statistical Package for the Social Sciences (SPSS) version 20 (IBM Corp., Armonk, NY) was used to analyze the data.

## Results

Descriptive statistics are presented in Table 1.

**Table 1:** Descriptive statistics

Variables (n = 49)		Mean ± SD/ Frequency
Age (years)		37.20 ± 10.05
Gender	Male	29 (59.2%)
	Female	20 (40.8)
Duration of injury (days)		2.43 ±1.768
Site of injury	Right	22 (44.9%)
	Left	27 (55.1%)
Hand Dominance	Right	37 (75.5%)
	Left	12 (24.5%)
Type of Fracture (AO Classification)	23-B3	4 (8.2%)
	23-C1	12 (24.5%)
	23-C2	19 (38.8%)
	23-C3	14 (28.6%)
Union Time (weeks)		11.98 ± 1.64
Radial Inclination (degree)		19.35 ± 2.15
Radial Shortening (mm)		5.20 ± 1.22
Volar Tilt (degree)		5.39 ± 0.95

The patient's mean age was 37.20 ± 10.05 years. Out of 49 patients, 29 (59.2%) were males, and 20 (40.8%) were females. The mean duration of the fracture time from the occurrence of injury to the presentation was 2.43 ± 1.768 days. The right hand was involved in 22 (44.9%) patients, and the left was involved in 27 (55.1%) cases. For hand dominance majority of the patients were right hand dominant, that is, 37patients constituting about 75.5% of patients in the study, and only 12(24.5%) of the patients were left hand dominant .with respect to fracture pattern (AO classification), most of the patients, that is, 19 patients were of type 23-C2, 14 patients belonged to AO type 23-C3, 4 (8.2%) patients sustained AO type 23-B3 type of fracture and 12 patients sustained AO type 23-C1 type of fracture. Union was achieved in almost all fractures except one case, which went into non-union despite the adequate initial reduction, and the mean time of union was 11.98 ± 1.64 weeks with a range over a minimum of ten-week and a maximum of 20 weeks. For radiological parameters, radial inclination, radial shortening, and volar tilt, the mean values at the six months for the above parameters were 19.35 ± 2.15, 5.20 ± 1.22, and 5.39 ± 0.95, respectively.

**Figure 1:**
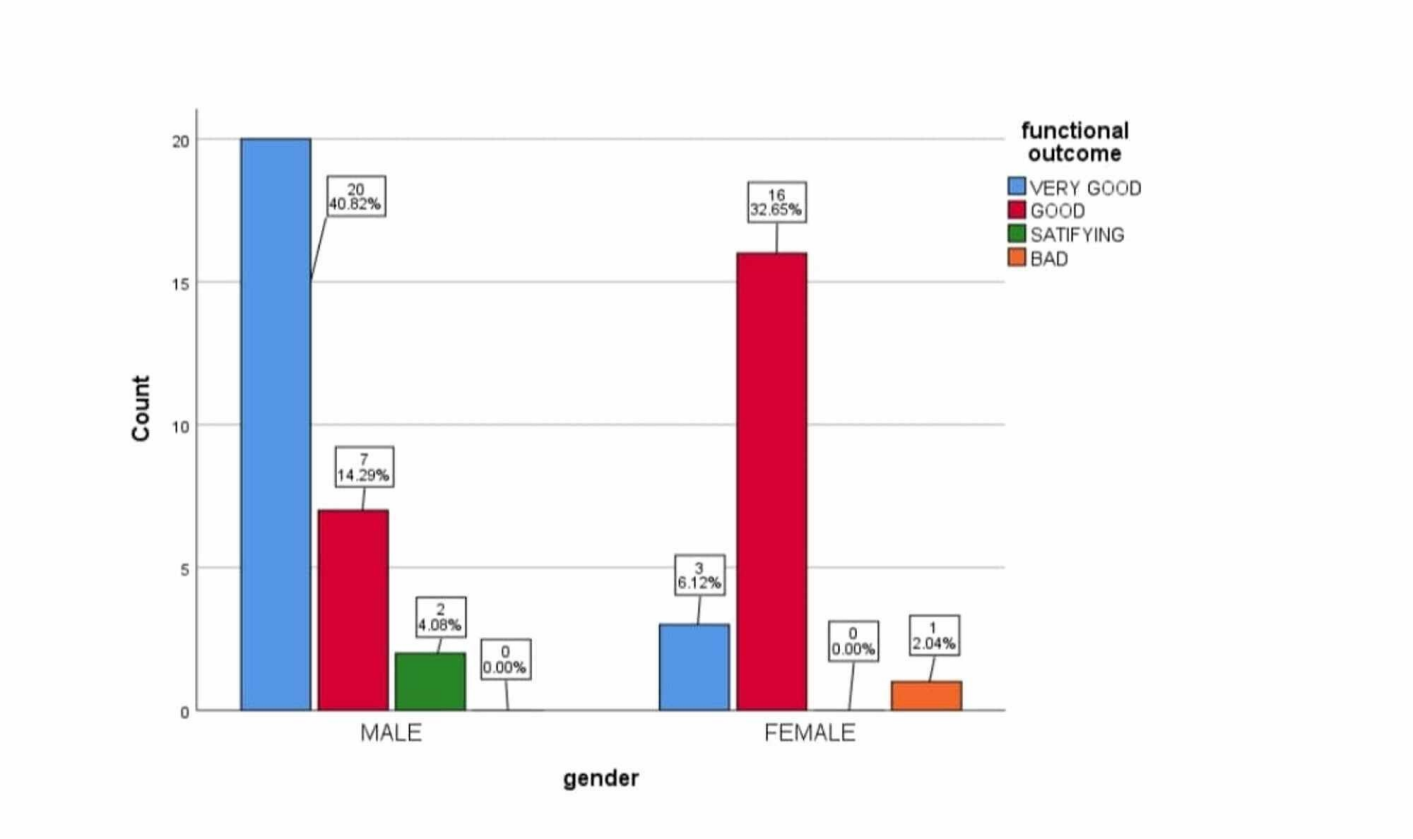
Stratification of functional outcome with gender

With respect to the stratification of functional outcome very good and good functional outcome was achieved in 46 patients (93.8%) in both the groups, the satisfactory outcome was achieved in two (4.1 %) cases and one patient had a bad outcome in which union was not achieved and went into non-union. Functional outcome of the fractures with respect to gender have been presented in Figure 1.

Concerning complications of the surgery, no complication was encountered in 44 (89.8%) of the cases, a superficial infection developed in three (6.1%) cases, wound dehiscence occurred in 1 (2%), and median nerve neuropathy developed in one (2%) patient as evident in Table 2.

**Table 2:** Post-operative complication

Complication	Frequency	Percentage
No complication	44	89.8%
Superficial infection	3	6.1%
Wound Dehiscence	1	2%
Median nerve neuropathy	1	2%
Total	49	100%

## Discussion

The distal end of the radius fractures is the most common fractures being treated, presenting to the emergency department. Though it is prevalent in the old age population due to osteoporosis, it’s also more commonly present in the young population due to high-velocity injuries [3]. Multiple opinions are there in terms of treatment of distal radial fractures from the conservative, that is, closed reduction and cast application to open reduction and internal fixation if the anatomical reduction and alignment is not achieved by the conservative means [12]. Intraarticular fractures of distal radius being the most complex injuries, various types of implants are used for the treatment of intraarticular fractures. Locking volar plates provided relatively stable construct in terms of rigidity, and it provides good stable alignment between the metaphysis and diaphysis, which might not be possible with conventional non-locking volar plates, dorsal plates or external fixation [13]. Volar locking plates have shown better results in comparison to non-locking volar and dorsal plates in biomechanical studies [14].

The mean age of the patients in our study noted was 37.20 ±10.05, whereas, in a study conducted by Kenny Kwan et al. [15], the mean age was 51 years. In another study conducted by Ansari et al. [16], the study's mean age was 39 years, which was almost comparable with our study. Males were most commonly affected in our study, comparable to the study conducted by other authors as well [17]. Though right-hand dominance was noticed in our study, the left side was most affected in about 27 (55.1%) cases compared to the right side, which was involved in 22 (44.9%) cases. Most fracture patterns in our study were type AO 23-C2 followed by23-C3, 23-C1, and 23-B3, respectively. The average time to union in our study was 11.98 ± 1.64, whereas in a similar type of study conducted by Arora et al. [18] and Orbay and Fernandez [19] showed union slightly earlier than our study at seven weeks. Concerning functional outcome in our study, we achieved in 93.2% of cases, a satisfactory outcome in 4.2% of cases, and bad outcomes in 2% of cases, comparable to studies conducted with other authors such as Kenny Kwan et al. [15] Showed excellent outcome in 88% of cases of a good outcome in 8% of cases.

Concerning complications in our study, 44 patients were free of any complications. Three patients developed superficial wound infection that was managed with an oral antibiotic and did not require any further intervention. Wound dehiscence was observed in 1 patient, which was managed by re-suturing and responded well to it. One patient in our study developed median nerve neuropathy after the surgical procedure, which was managed by the wait-and-watch policy, and it recovered completely by eight weeks. None of the patients in our study developed wrist joint or small joint of hand stiffness that remains the major problem with conservative treatment with the cast.

## Conclusions

Despite the multiple treatment options and approaches available for managing intraarticular distal radius fractures, volar locking plate fixation through the volar approach seems to be the better option in terms of decreased complication, stable reduction and fixation of the fracture, and association with good functional outcome in these fractures.
